# Machine learning with asymmetric abstention for biomedical decision-making

**DOI:** 10.1186/s12911-021-01655-y

**Published:** 2021-10-26

**Authors:** Mariem Gandouz, Hajo Holzmann, Dominik Heider

**Affiliations:** 1 Department of Data Science in Biomedicine, Faculty of Mathematics and Computer Science, University of Marburg, 35032 Marburg, Germany; 2 Department of Statistics, Faculty of Mathematics and Computer Science, University of Marburg, 35032 Marburg, Germany

**Keywords:** Medical data science, Machine learning, Classification, Diagnostics

## Abstract

Machine learning and artificial intelligence have entered biomedical decision-making for diagnostics, prognostics, or therapy recommendations. However, these methods need to be interpreted with care because of the severe consequences for patients. In contrast to human decision-making, computational models typically make a decision also with low confidence. Machine learning with abstention better reflects human decision-making by introducing a reject option for samples with low confidence. The abstention intervals are typically symmetric intervals around the decision boundary. In the current study, we use asymmetric abstention intervals, which we demonstrate to be better suited for biomedical data that is typically highly imbalanced. We evaluate symmetric and asymmetric abstention on three real-world biomedical datasets and show that both approaches can significantly improve classification performance. However, asymmetric abstention rejects as many or fewer samples compared to symmetric abstention and thus, should be used in imbalanced data.

**Supplementary Information:**

The online version contains supplementary material available at 10.1186/s12911-021-01655-y.

## Introduction

Machine learning (ML) and artificial intelligence (AI) have entered many areas of life and will also pave the way for a new era in biomedicine. These methods can improve medical treatment or diagnosis, identify novel subtypes, or provide new insights into survival prognostics. These methods consider all facets of data types, e.g., clinical health records, images, or omics data. Biomedical decision support systems based on ML and AI have entered many different studies and biomedical fields, e.g., Oncology [4], Pathology [10, 21, 35], Diabetes [6, 27], Human Genetics [20], and Infectious Diseases [14, 19, 28] as part of a growing trend towards precision medicine.

Overall, there is great potential for biomedical decision support systems based on ML and AI techniques and they have become key players in disease diagnostics, prognostics, and therapeutics [34]. However, biomedical datasets have very specific characteristics and suffer from many caveats regarding ML and AI. They often have a small number of samples and many parameters, a phenomenon called the small-*n*-large-*p* problem, missing values, and typically high class imbalance. Furthermore, biomedical decision support systems need to be probabilistically interpretable, typically addressed by calibration methods [1, 26]. While small-*n*-large-*p* and missing values are addressed by feature selection (also called biomarker discovery) approaches [23] and imputation techniques [29], the class imbalance is typically addressed by either down-sampling or data augmentation techniques. Moreover, uncertainty is critical when it comes to a medical decision. In contrast to human decision making, computational models typically make a decision also with low confidence. Machine learning with abstention better reflects human decision-making by introducing a reject option for samples with low confidence. The abstention intervals are typically symmetric intervals around the decision boundary. Thus uncertain predictions, i.e., predictions with low confidence or close to the decision boundary, are abstained when the consequences of the wrong classification can be severe, e.g., wrong treatment of a patient [15]. A symmetric abstention interval can be defined based on the prediction scores for biomedical decision support systems based on binary classification models. It is considered to be a range of test scores that is uncertain and does not necessitate a decision, and the test results are trichotomized into positive, negative, and undecided diagnoses.

Classifiers with abstention were first introduced by Chow [9] and further developed by Tortorella [30, 31]. Chow [9] derived a general error and reject trade-off relation for the Bayes optimum recognition system requiring the assumption of complete knowledge of the a priori probability distribution of the classes and the posterior probabilities (for instance, the distributions of the test results to be normal in both healthy and diseased subjects), which are usually not completely known in real-world problems. Thus, the reliance of this method on several assumptions represents an important limitation. Fumera et al. [12] demonstrated that Chow’s rule does not perform well if the a posteriori probabilities are affected by errors, suggesting the use of multiple reject thresholds, one for each class. The threshold is placed on the maximum a posteriori probability similar to Chow’s rule [8]). However, each class has a different threshold. Their results using nearest neighbor and neural network classifiers show that this approach outperforms the parametric assumption. Herbei and Wegkamp [15] developed excess risk bounds for the classification with a reject option setting where the loss function is the 0–1 loss, extended such that the cost of each reject point is $0 \le d \le 1/2$ (cost model). This approach generalizes the excess risk bounds of Tsybakov [32] for standard binary classification without rejection (which is equivalent to the case *d* = 1/2). This approach is further extended by Bartlett and Wegkamp [3] in various ways, including the use of the hinge loss function for efficient optimization. Nguyen et al. [24] developed an approach for abstention in multi-class problems based on pairwise comparison and integer programming, and separated epistemic, i.e., uncertainty caused by lack of information, and aleatoric uncertainty, i.e., due to intrinsic randomness. Very recently, Mortier et al. [22] developed a framework for Bayes-optimal prediction in multi-class problems, i.e., the subset of class labels with the highest expected utility. Campagner et al. [5] proposed a three-way-in and three-way-out approach, which is based on partially labeled data and abstention. They analyzed to what extent a classifier can make reliable prediction based on uncertain biomedical data.

While abstention intervals are typically considered to be symmetric, the goal of the current study is to show that asymmetric intervals are better suited for biomedical data, as these datasets are often imbalanced. We propose a simple, efficient, and novel method to optimally build an asymmetric type of abstaining binary classifiers using an asymmetric abstention interval around the intersection between the two distributions of positive samples (i.e., cases) and negative samples (controls) based on Pareto optimization, similar to the approach proposed by Herbei and Wegkamp [15].

## Methods

As a starting point, such a standard should satisfy the following requirements: (1) include information on the population providing the training data, in terms of data sources, cohort selection; (2) include training data demographics in a way that enables a comparison with the population the model is applied to; (3) provide detailed information about the model architecture and development so as to interpret the intent of the model and compare it to similar models and permit replication; and (4) transparently report model evaluation, optimization, and validation to clarify how local model optimization can be achieved and enable replication and resource sharing

### Data

We used three real-world biomedical datasets in our study covering different diseases/scenarios from different fields, namely oncology and reproduction. These datasets address breast cancer, prostate cancer, and cardiotocography to reflect different sample sizes and class imbalances. The datasets were collected from the UCI Machine Learning Repository [11]. The smallest dataset has 72 samples, and the largest dataset consists of 1831 samples. On average, the datasets have 540 samples, the median is 426, first and third quartiles are 124.5 and 713, respectively. The imbalance differs between 2.23 and 37.26% concerning the cases (i.e., positive class). On average, the imbalance is 18.42% (median is 17.7%), with the first and third quartiles at 9.78% and 28.49%, respectively. The number of features ranges from 3 to 32, on average 15 (median is 11), with the first and third quartiles of 9 and 21, respectively.

An overview of the datasets can be found in Table 1. We removed all samples and features with missing values. Thus the numbers may differ slightly from the original number of samples and features.

**Table 1:** Overview of the datasets

Name	Samples	Cases	Controls	Percentage	Features
wdbc	569	212	357	37.3	30
pc	376	225	151	59.8	9
ctg	1831	176	1655	9.6	22

Percentage represents the percentage of positive samples (i.e., cases) in the dataset. Features refers to the number of independent variables / features in the dataset

The Breast Cancer diagnostics dataset (wdbc) consists of 569 breast cancer patients [357 benign, 212 (37.3%) malignant] with 30 attributes. Features are computed from a digitized image of a fine needle aspirate (FNA) of a breast mass. They describe characteristics of the cell nuclei present in the image. All cancers and some of the benign masses were histologically confirmed. Cancer patients were given standard surgical and chemotherapy treatment. Adjunctive radiotherapy was given when indicated [33]. No missing attribute values.

The Prostate Cancer (pc) dataset consists of 376 samples [225 (59.8%) cancer, 151 (40.2%) controls] with nine features, e.g., age, race, and several clinical parameters [17].

The Cardiotocography dataset (ctg) consists of 1,831 fetal cardiotocograms, of which 1,655 are normal, and 176 have been classified as pathologic (9.6%). The dataset provides 22 features that have been calculated based on the cardiotocograms [2].

In Table 2, we report the data according to the MINIMAR standard to improve reproducibility [16].

**Table 2:** Data sets description

Study population and setting				
Name	Population	Study setting	Data source	Cohort selection
wdbc	Breast cancer patients	U.S. hospital	Digitized images	Adults
pc	Prostate cancer patients	U.S. hospital	EHR	Adults
ctg	Pregnant women	E.U. hospital	Fetal cardiotocograms	Unborn

Patient demographic characteristics				
Name	Age	Sex	Race	Ethnicity
wdbc	Not provided	100% female	Not provided	Not provided
pc	mean 66.04 y	100% male	90.45% white, 9.55% black	Not provided
ctg	Not provided	Not provided	Not provided	Not provided

### Implementation

All analyses have been carried out in Python v.3.8.5 with pandas (v.1.1.3), seaborn (v.0.11.0), Matplotlib (v.3.3.2), NumPy (v.1.19.2), scikit-learn (v.0.23.2), and Plotly (v.4.14.2).

### Machine learning

In supervised ML, the data is given by a training set

$${\mathcal {T}}=\left\{ \left( {\mathbf {x}}_{1}, y_{1}\right) , \ldots ,\left( {\mathbf {x}}_{m}, y_{m}\right) \right\} \subset ({\mathcal {X}} \times {\mathcal {Y}})^{m},$$

with *m* data pairs $(\mathbf {x_i}, y_i)$, where $\mathbf {x_i}$ is a vector of the observations for the *i*th data point and $y_i$ is its class label. The elements of the training set are called training data. ${\mathcal {X}}$ is called the feature space and the dimension *n* of this space corresponds to the number of features $X_1,\ldots ,X_n$, which are used to describe the observations. Hence ${\mathbf {x}}_{i}=\left( x_{i 1}, \ldots , x_{i n}\right) ^{\top } \in {\mathcal {X}}$ for *i* = 1, …, *m*, where $x_{ij}$ is the value of the feature $X_j (j=1,\ldots , N)$ in the *i*th observation. Furthermore, each observation $\mathbf {x_i}$ is associated with a class label $y_i \in {\mathcal {Y}}$, where ${\mathcal {Y}}$ denotes the set of possible labels (sample space for short). In the simplest case, the binary classification, the set ${\mathcal {Y}}$ consists of only two class labels, which are usually referred to as positive (cases) and negative (controls); or + 1 and 0.

The goal of supervised learning is thus to learn the relationships between the observations/features and the class label, based on the training set, to assign a class label to an observation as accurately as possible. Given the training set, the ML method learns a decision function (also called a classifier or model)

$$f: {\mathcal {X}} \rightarrow {\mathcal {Y}},$$

that performs classifications by mapping the observations from the feature space ${\mathcal {X}}$ to the sample space ${\mathcal {Y}}$ and reducing the error rate iteratively.

However, the decisions made by the model may be wrong for some instances, mainly when these instances are close to the binary decision border.

Thus, we extend the definition of a standard classifier, and we add a new label ®, which is referred to as abstaining: Given ${\mathcal {X}}$ and ${\mathcal {Y}}$ as defined above, an abstaining classifier is defined as a classifier which labels an instance $\mathbf {x_i} \in {\mathcal {X}}$ with an element from ${\mathcal {Y}} \cup \{{\textregistered }\}$.

In order to evaluate the performance of symmetric abstention and asymmetric abstention, we analyzed two scenarios, namely (1) the imbalanced data as it is and (2) down-sampled, balanced data.

To compare the different scenarios, we used the Logistic Regression (LR) as a base model. As the abstention procedure is independent of the ML method used, these results can be generalized for other ML models.

### Statistical evaluation

The LR models were trained and evaluated based on stratified hold-out validation and split into training and test data. We used 40% of the data as training data for the wdbc and pc datasets and 50% for training with the ctg dataset. We used the Matthews correlation coefficient (MCC)

$${\rm MCC}=\frac{{\rm TP} \cdot {\rm TN}-{\rm FP} \cdot {\rm FN}}{\sqrt{({\rm TP}+{\rm FP}) \cdot ({\rm TP}+{\rm FN}) \cdot ({\rm TN}+{\rm FP}) \cdot ({\rm TN}+{\rm FN})}}$$

with true positives (TP), true negatives (TN), false positives (TP) and false negatives (FN) to estimate the performance of the models as this metric has been shown to be particularly well-suited for imbalanced data [7].

In Tables 3 and 4, we report the model architecture and evaluation description according to the MINIMAR standard to improve reproducibility [16].

**Table 3:** Architecture description

Model architecture				
User	Task	Architecture	Features	Missingness
Clinicians	Prediction	Logistic regression	Documented and provided for all models in detail	Missing data were removed

**Table 4:** Evaluation description

Model evaluation			
Optimization	Internal validation	External validation	Transparency
Documented and provided for all models in detail	Stratified hold-out validation	Not performed	Data publicly available, code on request

### Symmetric and asymmetric abstention

The distributions of the test scores of controls (negative class) and cases (positive class) typically overlap in real-world scenarios, and as a result, there are both errors and correct decisions for the test scores between an upper (U) and lower (L) bounds within this range of overlap. In order to find the best symmetric and asymmetric abstention intervals for the test scores, i.e., the intervals that reduce wrong classifications but at the same time can classify most of the data, we used the maximum product of the MCC and the number of classified samples, i.e., samples outside the abstention interval.

**Fig. 1:**
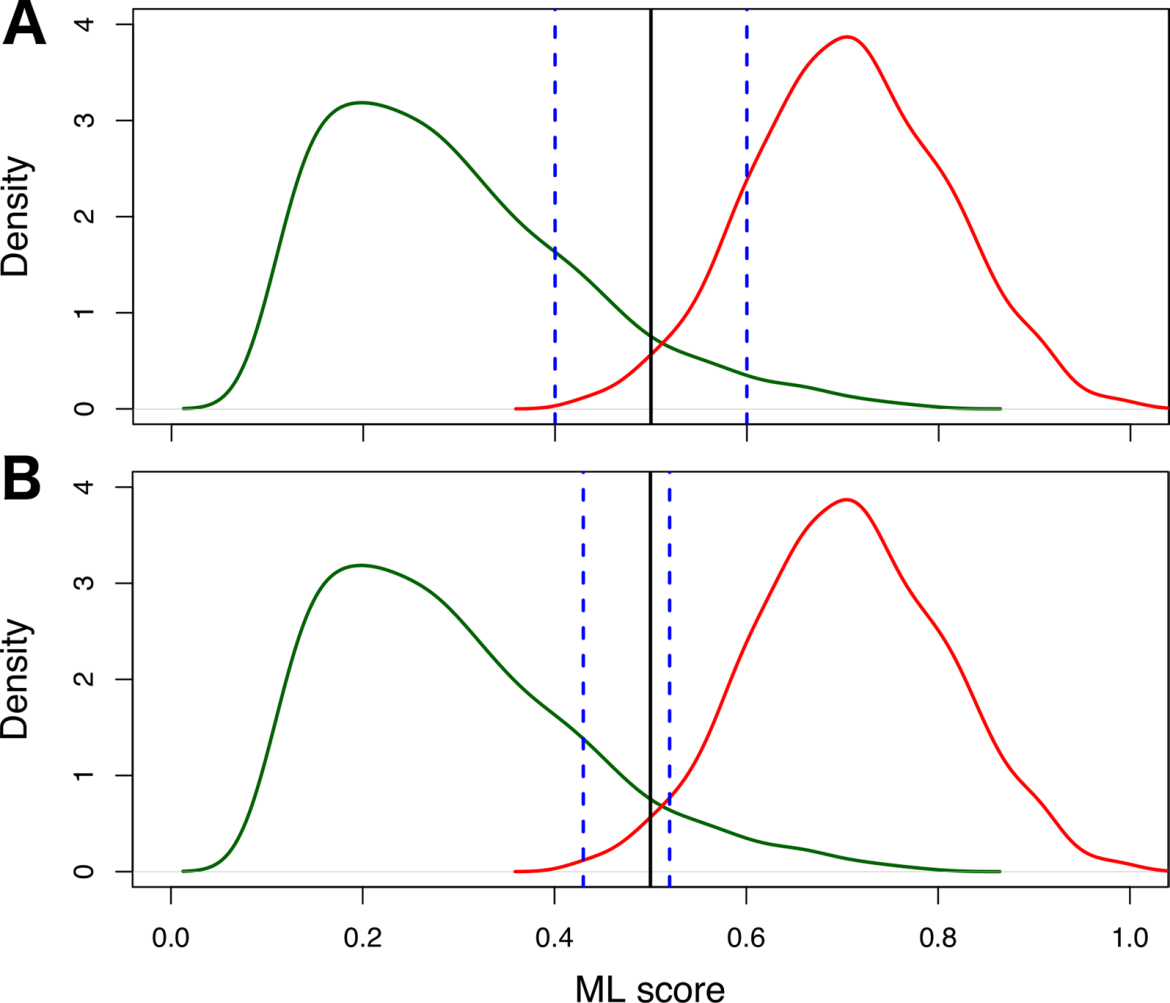
Symmetric and asymmetric abstention. **a** Symmetric abstention; **b** asymmetric abstention. The green curve represent the ML scores/probabilities of the controls (negative class), whereas the red curve represents the ML scores/probabilities of the cases (positive controls). The black vertical line marks the decision boundary and the blue dashed lines mark the borders of the abstention interval

#### Symmetric abstention

The upper and lower bounds of the symmetric abstention interval are defined with both curves as preliminary cut-point. The default is to use a threshold of 0.5 as the cut-off. Let us assume that $\Delta$ is the best width of the symmetric abstention interval. Thus, the symmetric abstention interval method will output $\Delta /2$ representing *best interval*. This means the best symmetric abstention interval is straightforward, simply requiring computing [*L*, *U*] with $L=0.5-{\it best\,interval}$ and $U=0.5+{\it best\, interval}$.

Furthermore, it is essential to note that the binary classifier needs to be already trained and give us the classes’ probabilities. Probabilistic interpretation is, for instance, possible for the logistic regression but may not be directly possible for other ML methods, e.g., deep neural networks or support vector machines. In order to provide probabilities for any ML model, calibration methods need to be employed [26].

The main functions for the symmetric abstention are fit() and cost(). fit() finds the value of the *best interval*. It takes test features (*X_test*), test labels (*y_test*), abstention interval minimum width (*low*), abstention interval maximum width (*high*), and the distance between two adjacent values at each incremental abstention level (*step*) as input. We set the parameters for the grid search as follows: $low =0$, $high =0.16$, and $step =0.01$. Starting at 0, which means no abstention interval at all and then going as high as 0.16. The values represent probabilities, thus, the maximum value can be 0.5 (i.e., 0.5 abstention at each side covers the whole probability range from 0 to 1). Max is set to 0.16 in order to stop at 0.15 taking a 1% abstention margin, which covers basically 2% as it goes from the left side and the right side of the threshold, and then 0.02, 0.03, etc. up to 0.15, which covers 30% of the range.

Next, the intervals are initialized the *MCC* and the *fractional size* of the samples (i.e., between 0 and 1; 0 corresponds to no data at all left, and 1 corresponds to all the data) are calculated. Each time the symmetric abstention interval grows, the size of the samples will decrease. Our two conflicting goals are maximizing the MCC while maximizing the size of the testing set that will be classified. We consider this problem as a Pareto optimization problem (also known as multi-objective optimization), where no single solution exists that optimizes each objective simultaneously, i.e., a problem for which there are many possible solutions from amongst which we want to find “the best”. Obviously, if we maximize for *mcc_values*, we cannot maximize for *sample sizes* and vice versa. After that, a possibly infinite number of Pareto optimal solutions are found. To overcome such problems and choose, we have to add additional subjective preference information and find a single solution that satisfies it. If no additional subjective preference information can be made, all Pareto optimal solutions are considered equally good. Thus, a set of Pareto optimal solutions will be generated and the best one can be selected according to the additional subjective preference as being the *score* = *mcc_values* $*$ *sizes*. We choose one of the obtained solutions using a simple one-dimensional grid search approach for function optimization.

The cost() function returns the *MCC* and the *fractional size* of the testing set. It takes as input the test features (*X_test*), test labels (*y_test*), and the chosen interval (*interval*), which is initialized with 0. After a sanity check, i.e., that $\hbox {interval} \in \{0,0.5\}$, the class probabilities of the test features are predicted and obtain the maximum probability for the samples. Any prediction that is not in the suitable range, which is {*maximum probability* − 0.5} (that is to center it), will be eliminated. All predictions that have an absolute value smaller than the interval are removed. All predictions with values larger than the interval are kept. Next, the size of the remaining samples is calculated, i.e., the test features and test labels that are outside the interval. These samples are predicted with the model, i.e., they are outside the abstention interval to obtain *MCC* and the corresponding number of samples (*fractional size*). The pseudocode for the symmetric abstention optimizer algorithm is shown in algorithm 1.

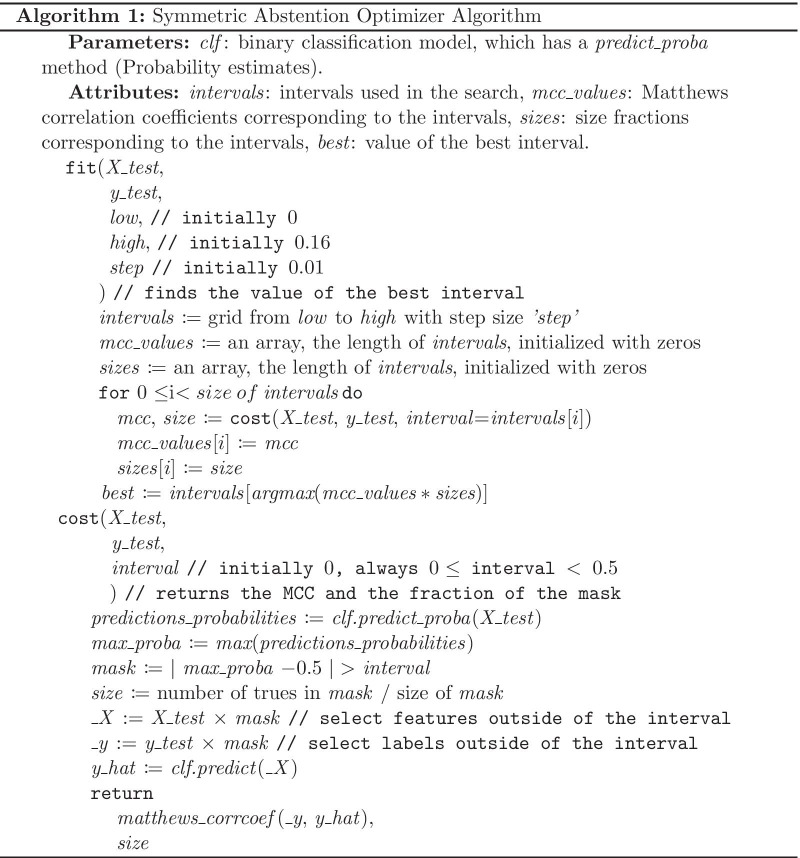



#### Asymmetric abstention

In contrast to the symmetric abstention interval method, in the asymmetric abstention interval method (see Figure 1) we have to search over the two parameters, namely the interval and also the anchor (i.e., the offset). The latter is basically the height of the interval, i.e., the center of the abstention line. The anchor and the interval are independent. Thus, the asymmetric abstention interval method will output the *best interval* and the *best anchor*, that maximize the output of the objective function argmax {(*mcc_values* * *sizes*)}. This means the best asymmetric abstention interval is straightforward, simply requiring computing [*L*, *U*] with *L* = 0.5 + *best anchor* − *best interval* and $U = 0.5 + {\it best \,anchor} + {\it best \,interval}$. The *best anchor* gets added to the center 0.5.

Thus, in this case, we create two variable ranges: the *intervals* and the *anchors*. Then we create *MCC*s and *sizes*, except that these are matrices now instead of arrays. To find the best combination, we use a two-dimensional grid search. We define the grid of the intervals and anchors that we want to search through, test each combination of possible parameters and select the best one for our asymmetric abstention interval. This approximation may be intractable in general since there would be infinitely many combinations to test for a continuous scale. The solution is to define a grid. This grid defines for each hyperparameter which values should be tested. In our case, where the intervals and the anchors are tuned: we could give the *intervals* the values between (0, 0.16) and the *anchors* the values between (− 0.2, 0.2). The hypothesis is that there is a specific combination of values of the different hyperparameters that will maximize the product of *MCC* and the *size* of the samples classified. So at each crossing point, the grid search will see what the maximum of the product *mcc_values* and *sizes* at this point is. After checking all the grid points, we know precisely which combination of parameters is the best. For the asymmetric abstention optimizer algorithm, we need some modifications of the core functions.

The fit() function in the asymmetric abstention interval method takes two additional parameters, namely (*width* and *num_anchors*), as input. We set them as follows: *width* = 0.2, and *num_anchors* = 20. The *anchors* start from − *width* to *width* with *num_anchors* steps. The cost() function in the asymmetric abstention interval method takes one additional parameter (*anchor*) as input, which is initialized with 0. Furthermore, the suitable range that we chose in the asymmetric abstention interval method is {*maximum probability* − 0.5 + *anchor*}. So we add the anchor to add the offset of the asymmetry (see algorithm 2).

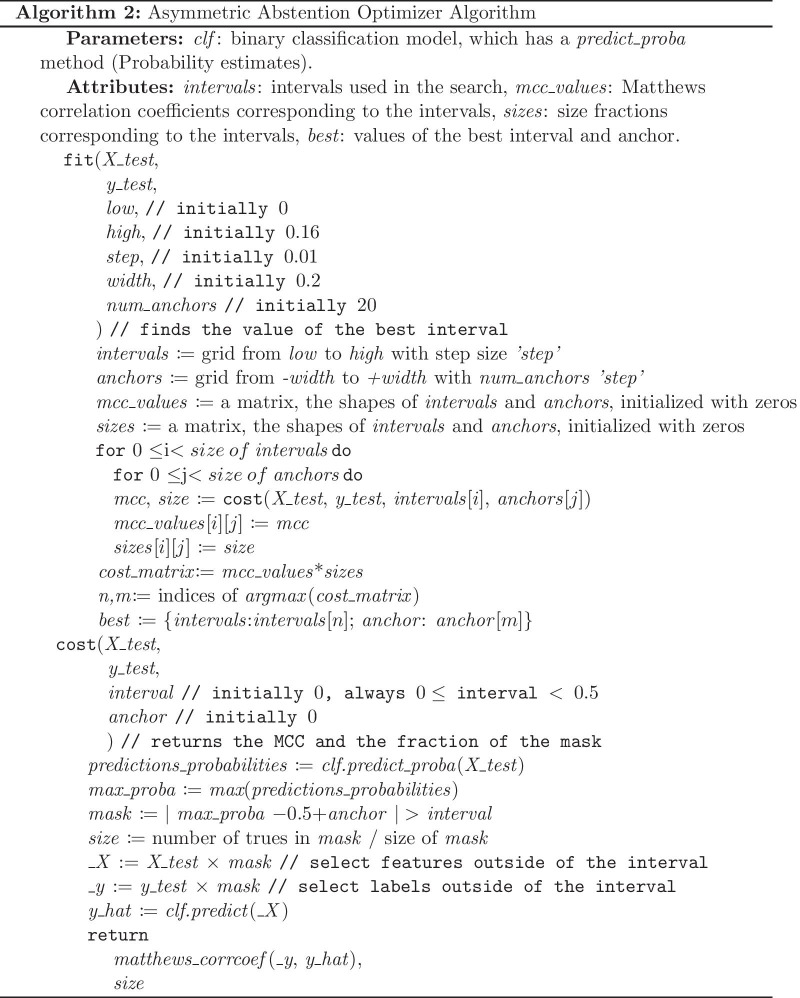



## Results

We evaluated the symmetric and asymmetric abstention with three real-world datasets. To this end, we analyzed two scenarios, namely (1) the imbalanced data as it is and (2) down-sampled, balanced data.

For the wdbc dataset, the LR model performed well on the imbalanced data with an MCC of 0.925. For the balanced design, the LR model produced a similar performance with an MCC of 0.93. For the pc dataset, the LR model reached an MCC of 0.483 on the imbalanced data and an MCC of 0.438 on the balanced data. For the ctg dataset, the LR model achieved very high MCC values, namely 0.963 for the imbalanced and 0.955 for the balanced design.

The corresponding confusion matrices and the probability distributions of the controls and cases for all three datasets and the two evaluated scenarios (imbalanced and balanced design) can be found in Additional file 1: Figs. S1–S6.

**Fig. 2:**
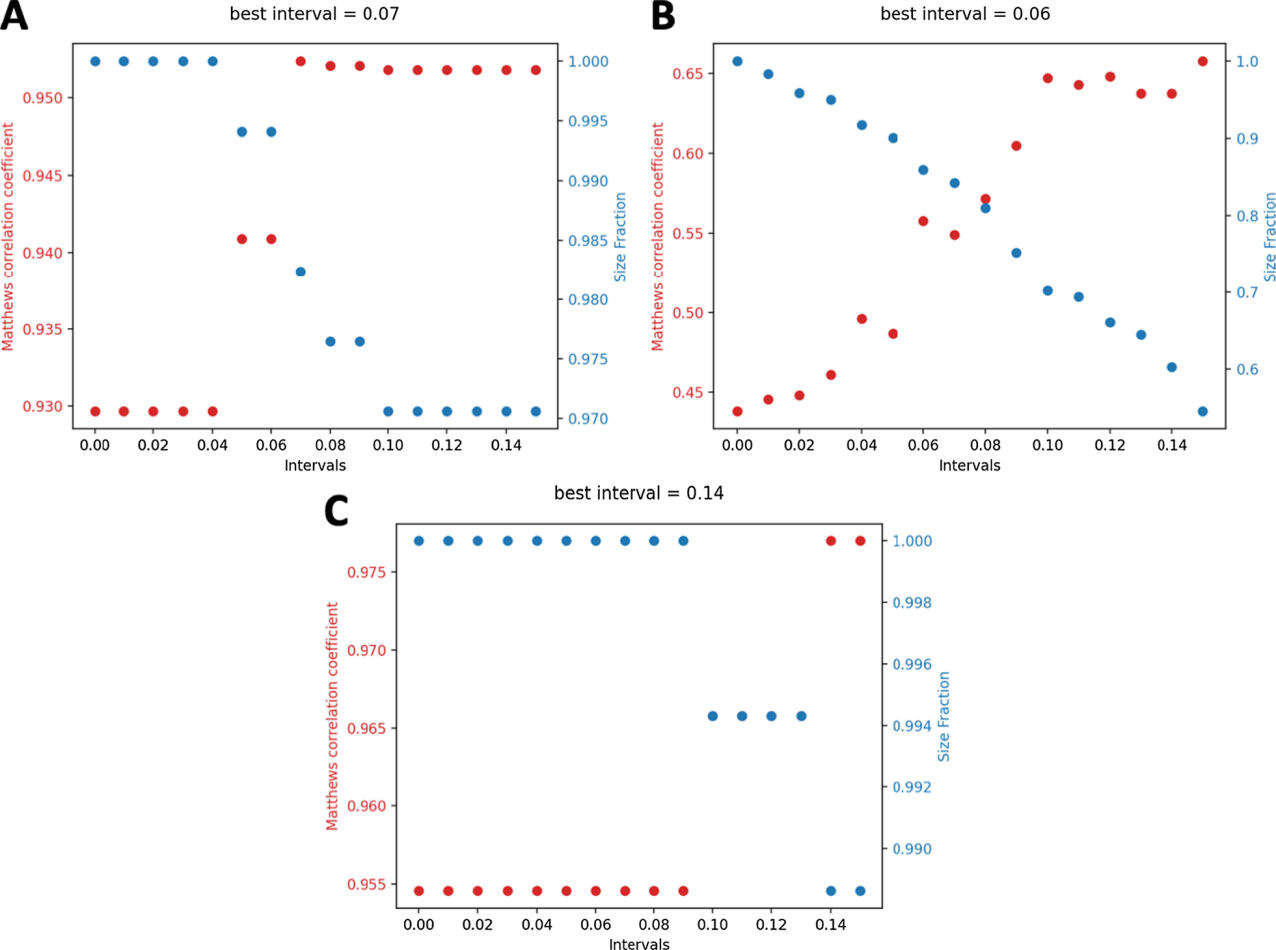
Symmetric abstention with balanced data. **a** wdbc; **b** pc; **c** ctg. The red and blue dots mark the MCC and size fraction, respectively, for a given interval

We next evaluated and compared the performance of the symmetric and asymmetric abstention. The symmetric abstention performs well on balanced data and can significantly increase the MCC for all datasets. For instance, for the wdbc dataset, the best interval is [0.43, 0.57] with an MCC of 0.952 and a size fraction of 98.2% (see Figure 2A). For the pc dataset, the symmetric abstention on the balanced data performed best for an interval [0.44, 0.56] with an MCC of 0.558 and a size fraction of 86% (see Figure 2B). For the ctg dataset, the symmetric abstention was best with an interval [0.36, 0.64], resulting in an MCC of 0.977 and a size fraction of 98.9% (see Fig. 2c).

On imbalanced data, the symmetric abstention reaches an MCC of 0.951, 0.556, and 0.975 with corresponding size fractions of 97.4%, 87.4%, and 99.6% for the datasets wdbc, pc, and ctg, respectively. The symmetric abstention can improve the MCC significantly. However, this comes with a rejection rate of up to 12.6%.

**Fig. 3:**
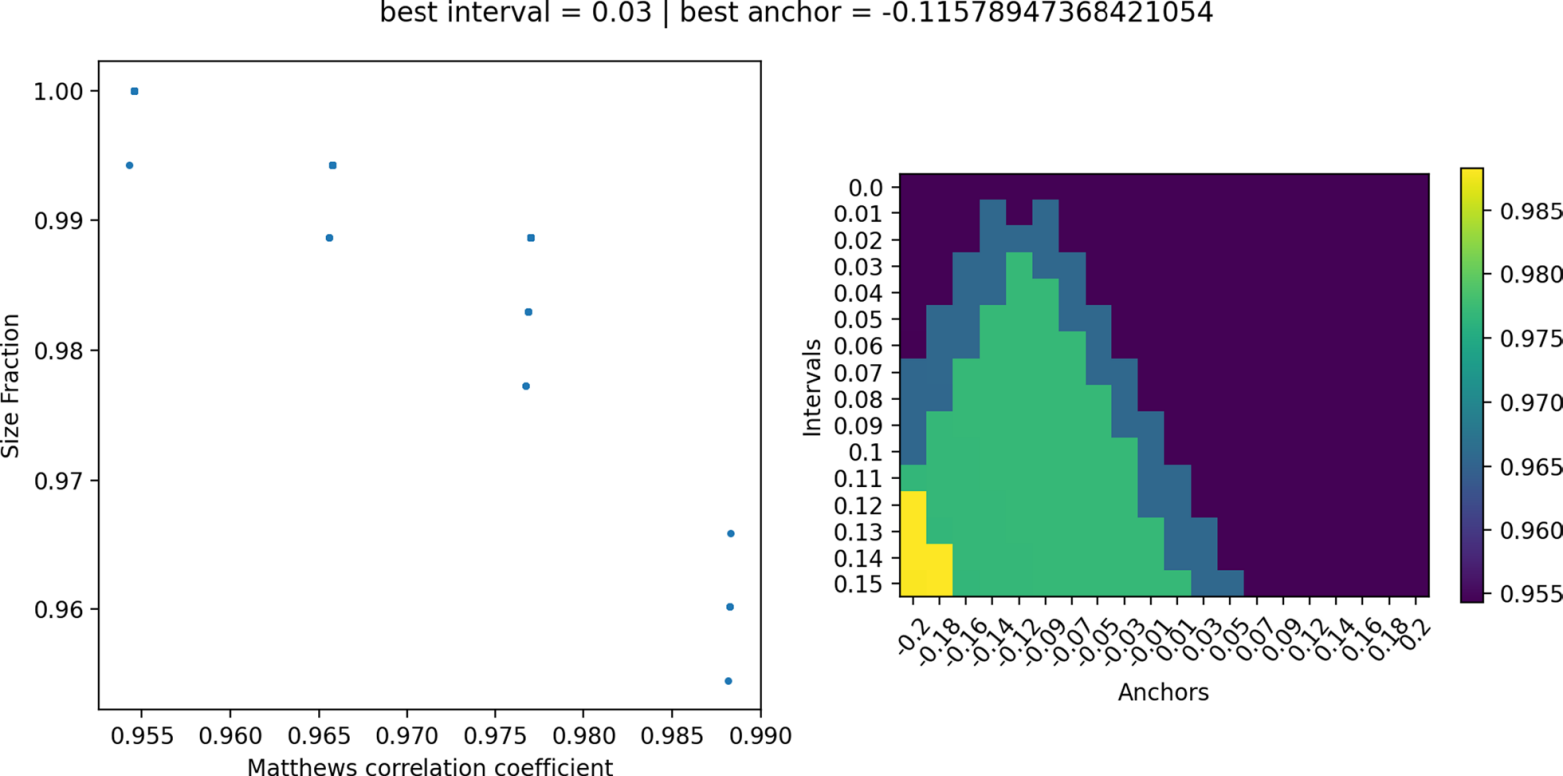
Asymmetric abstention for the ctg dataset. The scatterplot shows the MCC versus size fraction and the heatmap shows the MCC for each anchor in each interval

In contrast, the asymmetric abstention performs equally well in MCC for all imbalanced datasets, namely 0.98, 0.54, and 0.987 for the datasets wdbc, pc, and ctg, respectively. The rejection rate is similar to the rejection rate of the symmetric abstention approach with 4.3%, 7%, and 0.8%. However, it is always lower than 10%, which is not the case for symmetric abstention. In Figure 3, the asymmetric abstention is exemplarily shown for the ctg dataset. The corresponding figures for the wdbc and pc datasets can be found in the Additional file 1: Figs. S7 and S8, respectively. All results of the imbalanced and balanced analyses are summarized in Tables 5 and 6.

**Table 5:** Results of the models on the imbalanced data

Name	Abstention	MCC	Rejected	Interval
wdbc	None	0.925	–	–
wdbc	Symmetric	0.951	< 5%	[0.37, 0.63]
wdbc	Asymmetric	0.98	< 5%	[0.263, 0.463]
pc	None	0.483	–	–
pc	Symmetric	0.556	< 10%	[0.44, 0.56]
pc	Asymmetric	0.54	< 10%	[0.427, 0.467]
ctg	None	0.963	–	–
ctg	Symmetric	0.975	< 1%	[0.39, 0.61]
ctg	Asymmetric	0.987	< 1%	[0.252, 0.432]

Abstention represents the abstention approach, i.e., none, symmetric, or asymmetric abstention. Rejected refers to the percentage of rejected samples, and Interval represents the bounderies of the abstention interval

**Table 6:** Results of the models on the balanced data

Name	Abstention	MCC	Rejected	Interval
wdbc	None	0.93	–	–
wdbc	Symmetric	0.952	< 5%	[0.43, 0.57]
wdbc	Asymmetric	0.952	< 5%	[0.427, 0.467]
pc	None	0.438	–	–
pc	Symmetric	0.558	< 10%	[0.44, 0.56]
pc	Asymmetric	0.558	< 10%	[0.439, 0.539]
ctg	None	0.955	–	–
ctg	Symmetric	0.977	< 5%	[0.36, 0.64]
ctg	Asymmetric	0.955	< 1%	[0.354, 0.414]

Abstention represents the abstention approach, i.e., none, symmetric, or asymmetric abstention. Rejected refers to the percentage of rejected samples, and interval represents the bounderies of the abstention interval

## Discussion

Our study demonstrates that both the symmetric and asymmetric abstention can improve the MCC for real-world classification, thereby improving the diagnostic value of an AI model in medical applications. However, this does not come without costs. In order to improve the MCC, the samples are rejected, which is particularly significant for the symmetric abstention on imbalanced data. In our study, we analyzed different datasets with different degrees of imbalance from moderate to high imbalance. Asymmetric abstention is particularly useful and superior for imbalanced data compared to symmetric abstention. Thus, asymmetric abstention should be considered when the dataset is imbalanced, which is regularly the case in medical datasets. Moreover, abstention is particularly useful for machine learning in automated processes to reduce costs for healthcare, in particular time and costs for medical staff. Thus, abstention in healthcare can be used for instance in screening processes to reduce the number of diagnoses with a human expert in the loop. In the future, we will extend our approach to general multi-class problems (i.e., classification tasks with more than two classes) with a reject option. Although it is just an extension of binary classification, it is more challenging for the algorithms to be effective. The more classes to predict, the more complex the problem will be. Our results show the usefulness and applicability of asymmetric abstention. However, there is room for improvements since our method does not solve all the problems associated with medical diagnostic decisions based on test scores [18], and we do not suggest that it replaces the use of the symmetric abstention interval method in general. In the future, we intend to analyze the interplay between data augmentation and abstention as well as the interplay between calibration methods for probabilistic interpretation and abstention intervals. Calibration can be used to make machine learning scores probabilistically interpretable and thus transform the original score distribution into a probability distribution, which directly affects the abstention interval. Moreover, we did not address at all the relevant regulatory requirements in our approach to be considered as software as a medical device (SAMD) [13, 25]. However, our new method can be a good starting point to improve diagnostic software for biomedical decision-making.

## Supplementary Information

**Additional file 1**. Supplementary Figures 1–8.

## Data Availability

All datasets are publicly available. The Breast Cancer diagnostics dataset (wdbc) can be found at https://archive.ics.uci.edu/ml/datasets/Breast+Cancer+Wisconsin+%28Diagnostic%29, the Prostate Cancer (pc) dataset can be found in the R package lbreg https://cran.r-project.org/web/packages/lbreg/, and the Cardiotocography dataset (ctg) can be found at https://archive.ics.uci.edu/ml/datasets/Cardiotocography.
