# Supplementary material for: Machine learning with asymmetric abstention for biomedical decision-making
Source: BMC Med Inform Decis Mak. 2021 Oct 26;21:294. doi: 10.1186/s12911-021-01655-y (PMC8549182; doi:10.1186/s12911-021-01655-y)
Supplement: Supplementary file 1 — Additional file 1. Supplementary Figures 1–8. [file 12911_2021_1655_MOESM1_ESM.pdf]

Machine Learning with Asymmetric Abstention for Clinical Decision Support

Mariem Gandouz, Hajo Holzmann, and Dominik Heider

Supplement

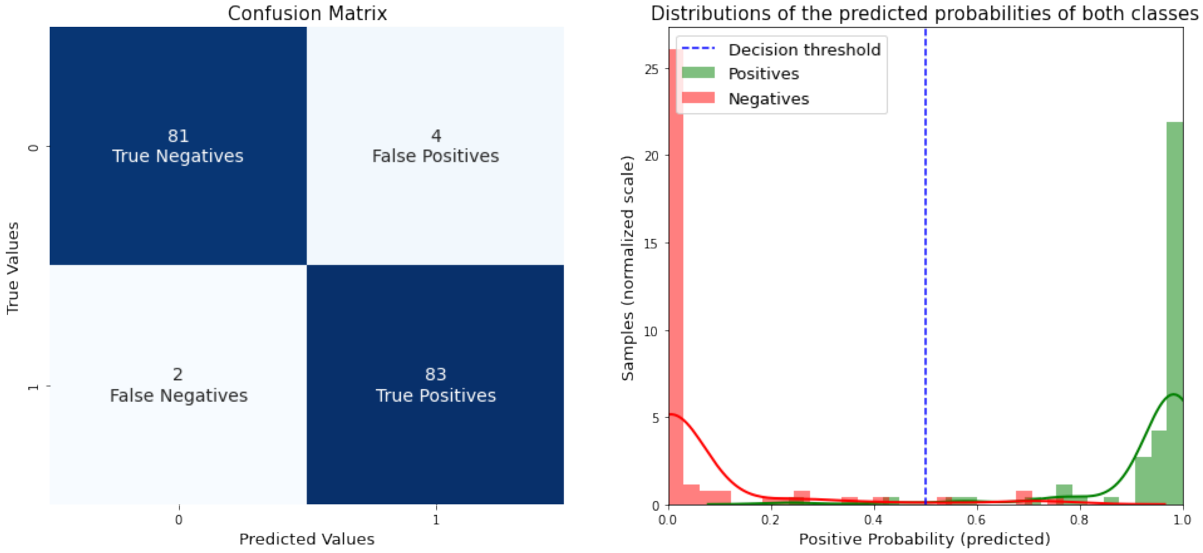

Figure 1: Confusion matrix and probability distributions for the balanced wdbc dataset.

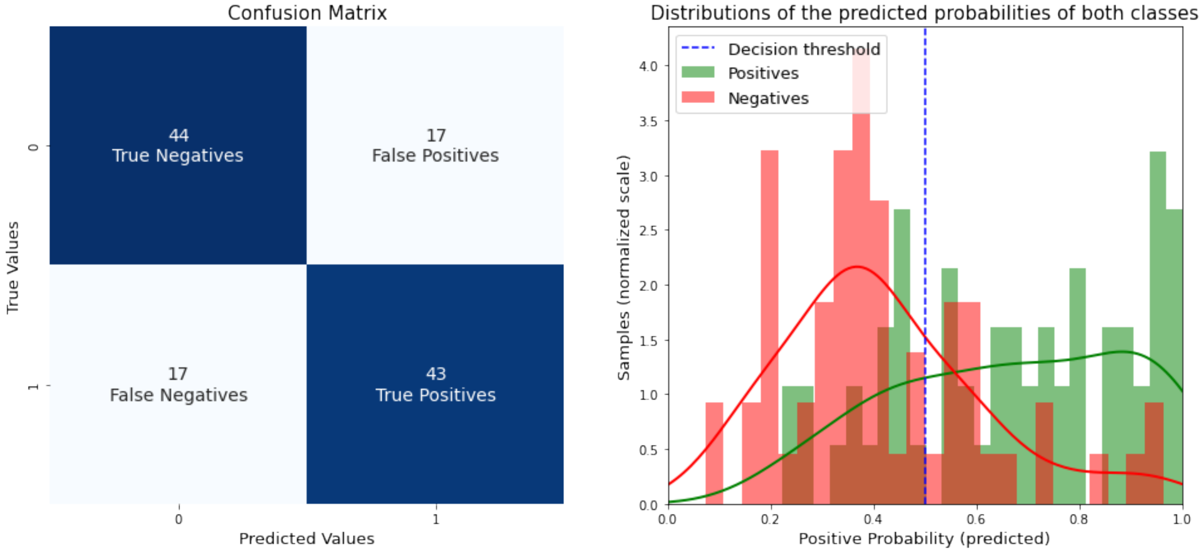

Figure 2: Confusion matrix and probability distributions for the balanced pc dataset.

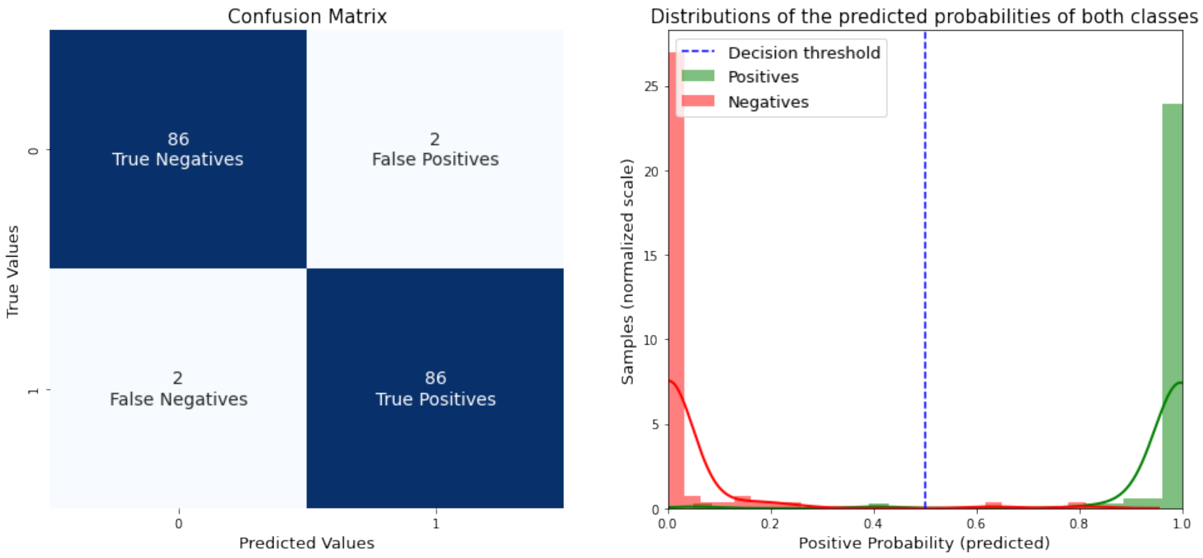

Figure 3: Confusion matrix and probability distributions for the balanced ctg dataset.

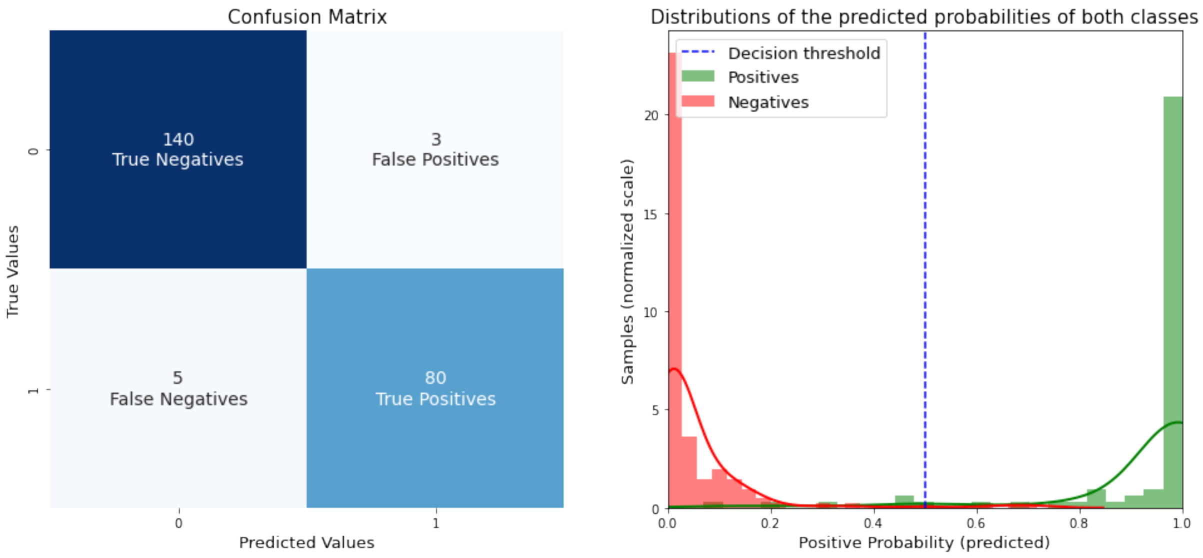

Figure 4: Confusion matrix and probability distributions for the imbalanced wdbc dataset.

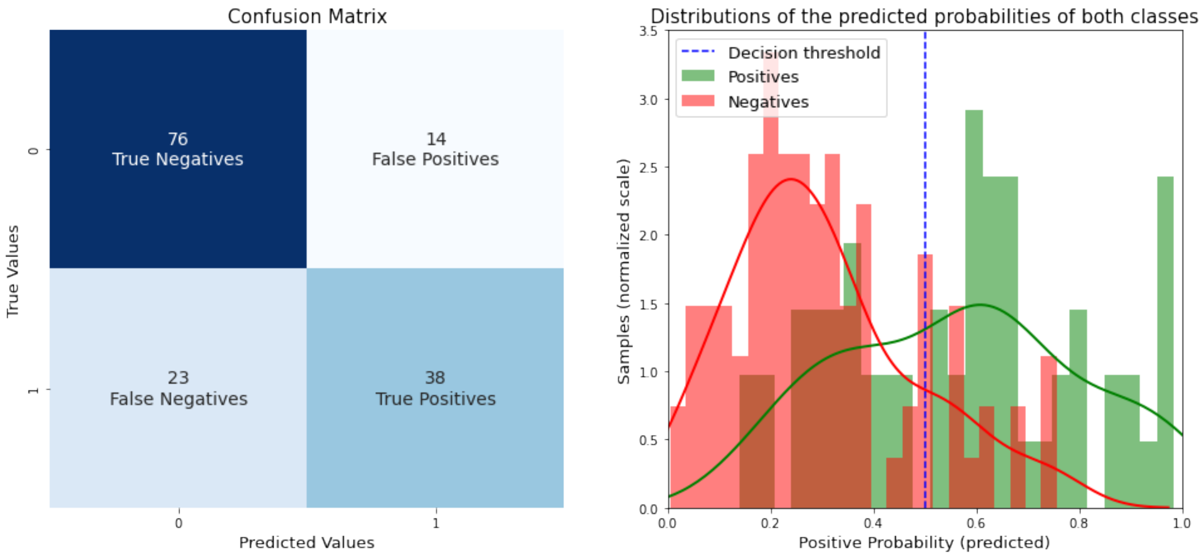

Figure 5: Confusion matrix and probability distributions for the imbalanced pc dataset.

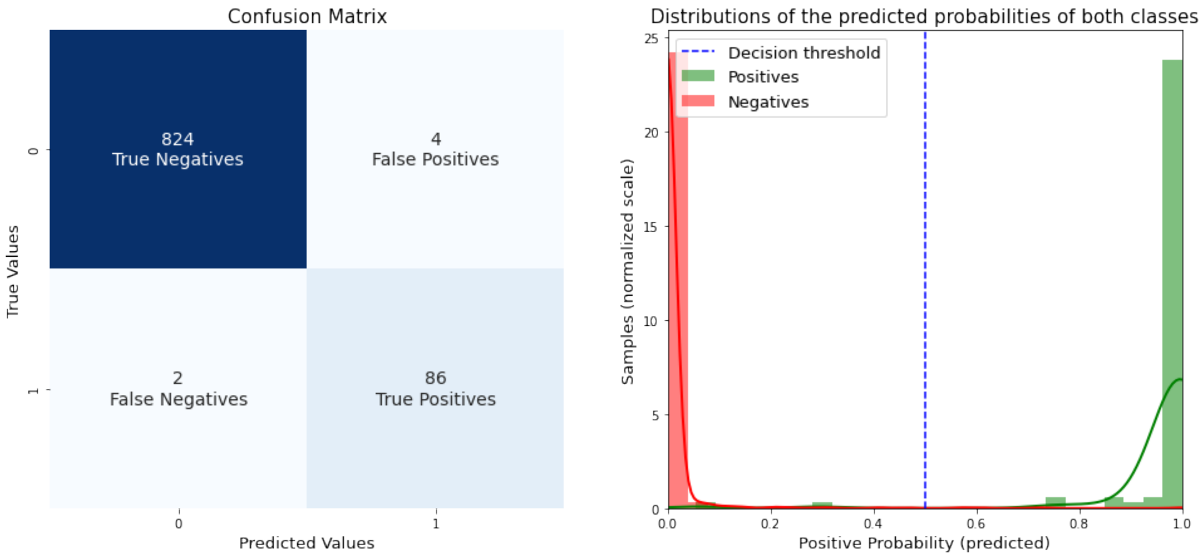

Figure 6: Confusion matrix and probability distributions for the imbalanced ctg dataset.

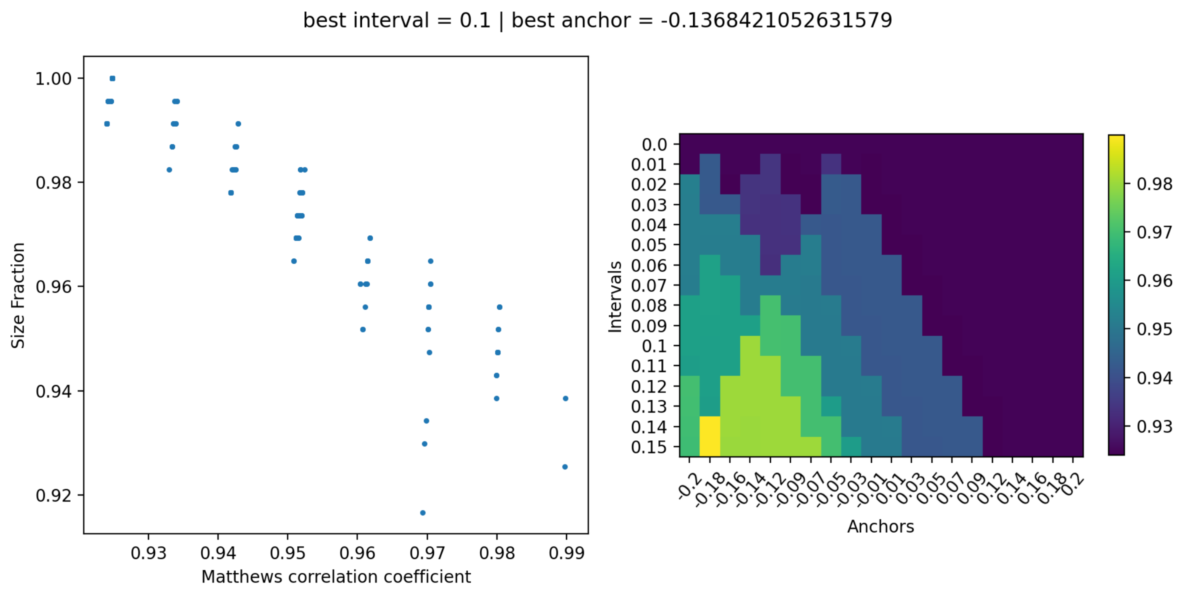

Figure 7: Asymmetric abstention for the wdbc dataset. The scatterplot shows the MCC versus size fraction and the heatmap shows the MCC for each anchor in each interval.

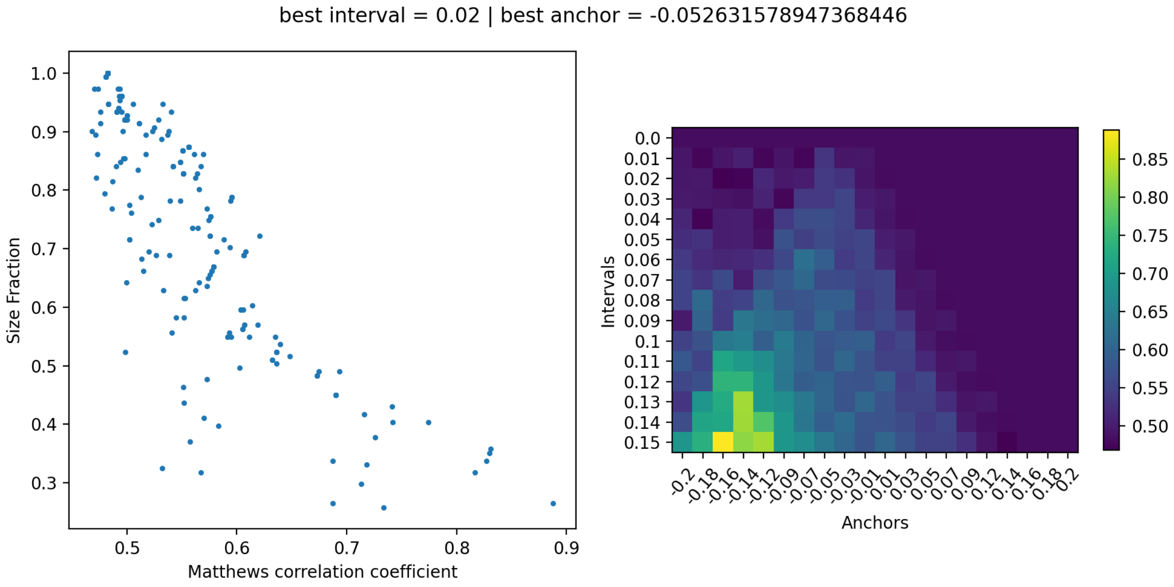

Figure 8: Asymmetric abstention for the pc dataset. The scatterplot shows the MCC versus size fraction and the heatmap shows the MCC for each anchor in each interval.
